# Diagnostic accuracy of deep learning for evaluation of C-spine injury from lateral neck radiographs

**DOI:** 10.1016/j.heliyon.2022.e10372

**Published:** 2022-08-24

**Authors:** Arunnit Boonrod, Artit Boonrod, Atthaphon Meethawolgul, Prin Twinprai

**Affiliations:** a Department of Radiology, Khon Kaen University, Khon Kaen, 40002, Thailand; b Department of Orthopedics, Khon Kaen University, Khon Kaen, 40002, Thailand; c AI and Informatics in Medical Imaging (AIIMI) Research Group, Faculty of Medicine, Khon Kaen University, Khon Kaen, 40002, Thailand

**Keywords:** Cervical spine injury, Lateral radiographs, Cervical spine CT, Deep learning

## Abstract

**Background:**

Traumatic spinal cord injury (TSI) is a leading cause of morbidity and mortality worldwide, with the cervical spine being the most affected. Delayed diagnosis carries a risk of morbidity and mortality. However, cervical spine CT scans are time-consuming, costly, and not always available in general care. In this study, deep learning was used to assess and improve the detection of cervical spine injuries on lateral radiographs, the most widely used screening method to help physicians triage patients quickly and avoid unnecessary CT scans.

**Materials and methods:**

Lateral neck or lateral cervical spine radiographs were obtained for patients who underwent CT scan of cervical spine. Ground truth was determined based on CT reports. CiRA CORE, a codeless deep learning program, was used as a training and testing platform. YOLO network models, including V2, V3, and V4, were trained to detect cervical spine injury. The diagnostic accuracy, sensitivity, and specificity of the model were calculated.

**Results:**

A total of 229 radiographs (129 negative and 100 positive) were selected for inclusion in our study from a list of 625 patients with cervical spine CT scans, 181 (28.9%) of whom had cervical spine injury. The YOLO V4 model performed better than the V2 or V3 (AUC = 0.743), with sensitivity, specificity, and accuracy of 80%, 72% and 75% respectively.

**Conclusion:**

Deep learning can improve the accuracy of lateral c-spine or neck radiographs. We anticipate that this will assist clinicians in quickly triaging patients and help to minimize the number of unnecessary CT scans.

## 1 Introduction

Traumatic spinal cord injury (TSI) is a leading cause of morbidity and mortality worldwide, the most common causes being traffic accidents and falls [1, 2]. Cervical spine fractures, which occur commonly in traumatized patients, might have devastating neurological implications. Instability and impingement of the underlying spinal cord can also occur as a result of these injuries [3, 4, 5, 6].

The most common locus of spinal cord damage is the cervical spine, with damage occurring at this level in 55% of all spinal cord injuries [7]. After a vertebral fracture, functional loss and neurological deficiencies can develop, and a delayed diagnosis might worsen the prognosis or necessitate invasive surgery [8]. Early identification and treatment are vital in preventing morbidity and mortality, as well as improving patient quality of life. Imaging plays a crucial role in this process, as damage to the patient's important neurological structures can be reduced by prompt and proper management—from diagnosis to therapy [9].

Plain cervical spine radiography is an important and commonly utilized tool in the evaluation of cervical spinal fractures. Anteroposterior and lateral cervical spine radiographs are often acquired in acutely traumatized patients as soon as they arrive in the emergency room, allowing for earlier detection of and intervention targeting many significant injuries [10, 11]. However, plain cervical spinal radiographs are insufficient to completely examine the cervical spine following forceful trauma, necessitating the use of a supplemental CT scan [12, 13]. In addition, standard 3-view radiography may be unreliable, missing up to 53% of all cervical spine fractures and producing perfectly normal results in up to 8% of patients with bony cervical spine injuries [14].

There has been great effort to reduce the number of unnecessary scans, including the use and implementation of the National Emergency X-Radiography Utilization Study (NEXUS) criteria and The Canadian C-Spine Rule (CCR) to reduce the number of unnecessary cervical spinal non-contrast CT scans [14, 15], as these evaluation methods are time-consuming, costly, and not always available in the primary care setting.

Imaging identification based on deep learning is a potentially effective diagnostic strategy that can reduce the burden that increased imaging places on radiologists, who must labor to maintain diagnostic accuracy and efficiency [16].

In this study, we analyzed cervical spine injury on lateral radiographs, the most readily available screening tool, using deep learning with the aim of enhancing their utility in a cost-effective manner.

## 2 Materials and methods

The local institutional ethics committee approved this retrospective analytical study with a waiver of informed consent. Office of The Khon Kaen University Ethics Committee in human research KKU EC approved this study with trial number “HE641351”.

### 2.1 Data collection

A list of patients who underwent a CT scan of the cervical spine from May 2015 through May 2020 was retrospectively acquired from our radiology reporting system. Basic patient characteristics, presence of lateral neck or lateral cervical spine radiographs, indications for CT scan, and CT reports were recorded. If lateral neck or lateral cervical spine radiographs were available, they were obtained from our picture archiving and communication systems (PACS) as JPEG, PNG, and BMP image files. All annotations in the images were removed.

Indications for CT scan were recorded and categorized based on the CCR and NEXUS criteria which includes age ≥65 years, dangerous mechanism (fall from elevation >3 feet, axial load to the head, or high-speed motor vehicle, motorized recreational vehicle, or bicycle collision), paresthesia in extremities, focal neurologic deficit, altered level of consciousness, and midline spinal tenderness.

Inadequate radiographs and those with evidence of previous surgery were excluded. Negative radiographs considered adequate were those with coverage from C1 to C7. Adequate positive radiographs were those covering the reported fracture/injury (e.g., radiographs that cover C1–C5 would be considered adequate if the reported injury was at C3).

### 2.2 Ground truth and labeling

**Figure 1:**
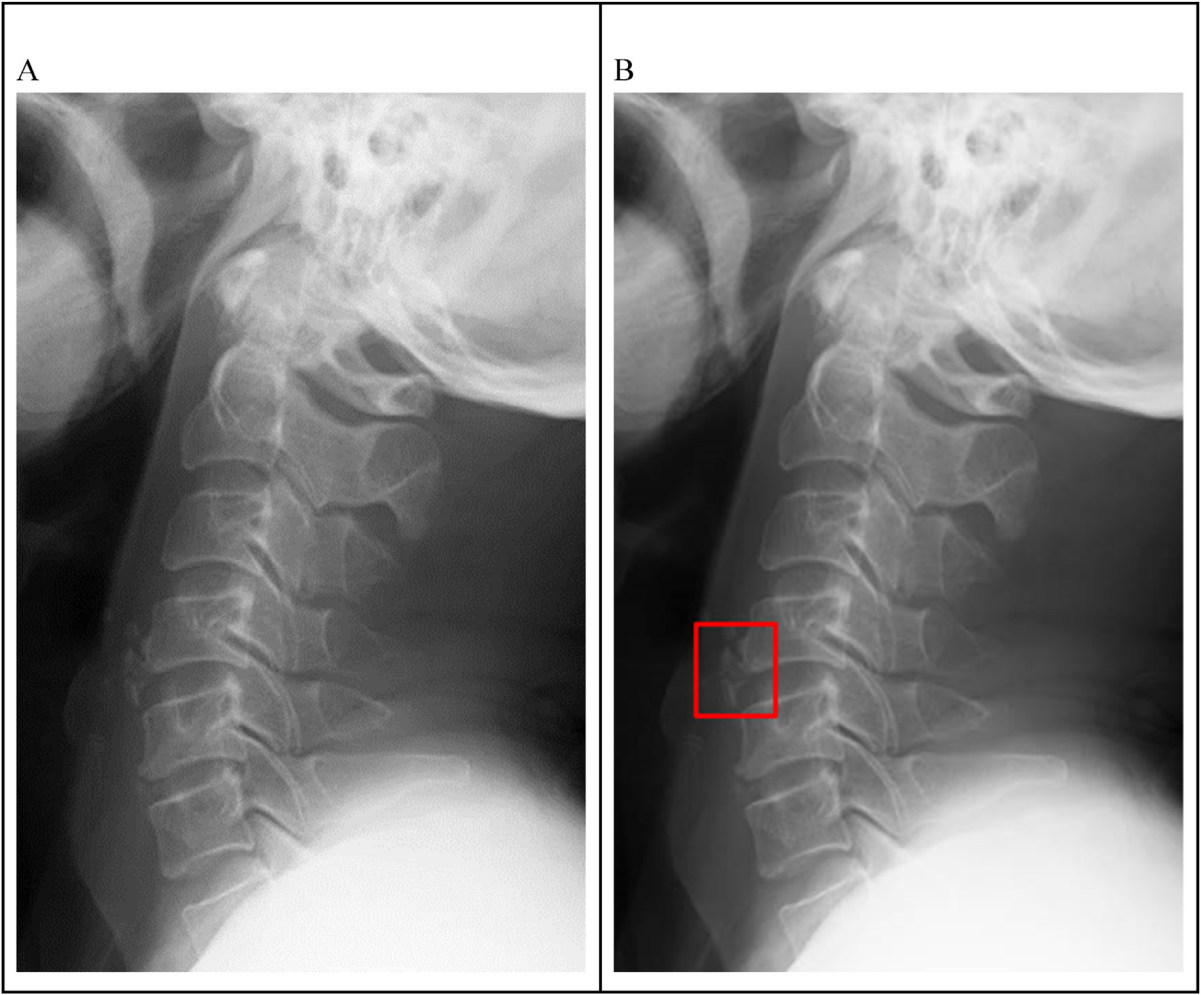
Lateral cervical radiograph with a CT report of C4/5 osteophyte fracture. The fracture is visible on the radiograph and was labeled accordingly.

**Figure 2:**
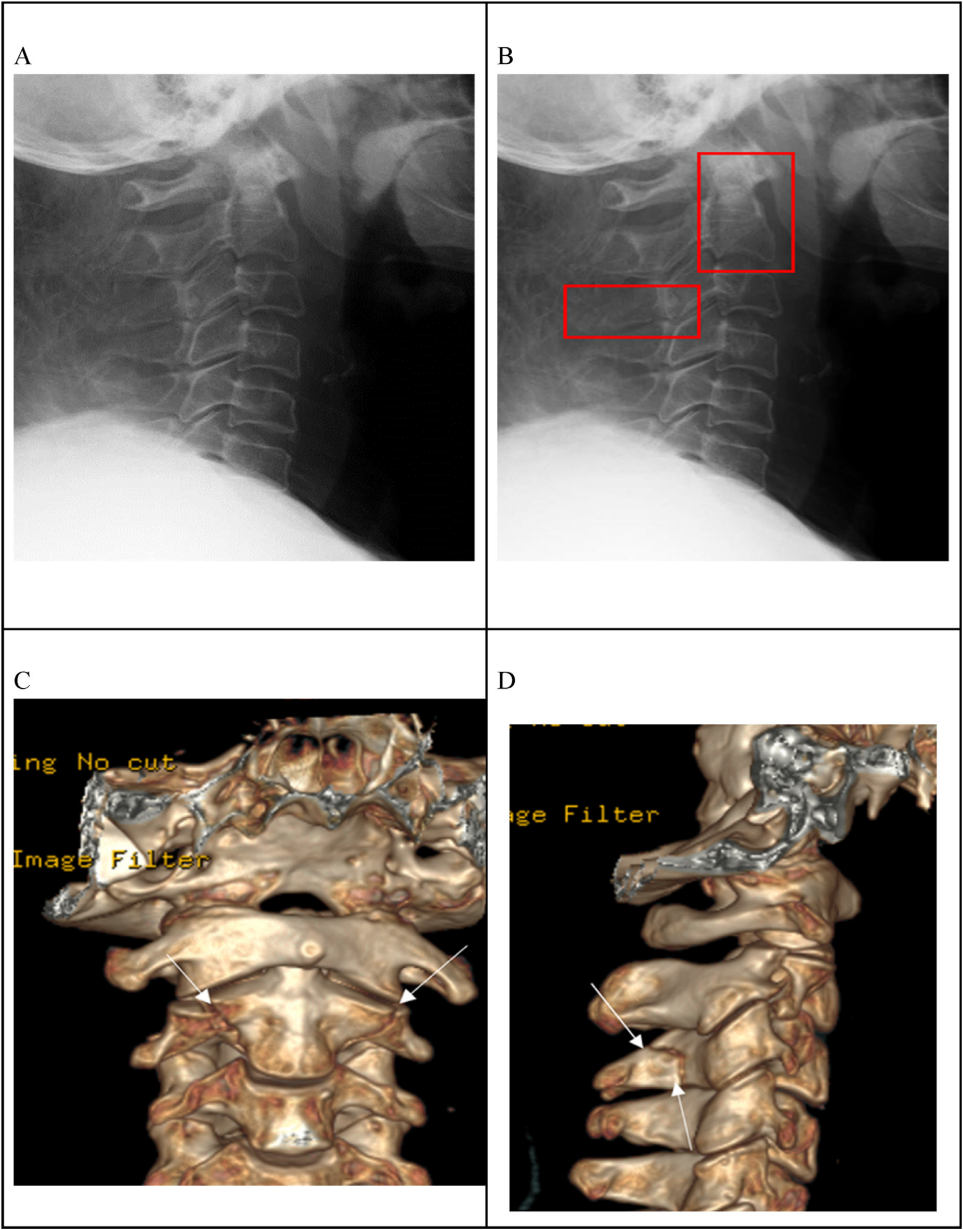
Lateral cervical radiograph with a CT report of fracture at C2 vertebral body and fracture of C3 transverse process. The fractures are not clearly visible on the radiograph (A) and were labeled at the relevant anatomy(B). (C) and (D) 3D CT scan of this patient shows fractures at C2 vertebral body and C3 spinous process.

The results from the CT scans were used as a ground truth. Radiographs with negative CT reports were labeled as negative. Those with positive reports were labeled with a bounding box by a neuroradiologist with 5 years’ experience. If the injury was visible on plain radiographs, the label would be placed at that area. If the injury was not clearly visible, the label would be placed at the relevant anatomical location (Figures 1 and 2). Twenty negative and 20 positive radiographs were randomly selected for use as a test set.

### 2.3 Deep learning network configuration

Three YOLO network models (version 2, version 3, and version 4 algorithms) were used to detect and classify cervical spine injury. The study was performed using a high-performance computer system running Windows 10. All the experiments were conducted on the server with the following configuration: CPU i510210U (1.6 GHz), RAM 16 GB, GPU UHD (8 GB), and NVIDIA GeForce MX 250 (2 GB). Programming languages used were C++ and Python.

Data augmentation was performed using -20 to 20-degree rotation and enhanced image contrast. No Gaussian noise or blur conditions were applied because we concluded these would cause deterioration to the injury visualization. The augmentation process provided 4,500 images for model training.

**Figure 3:**
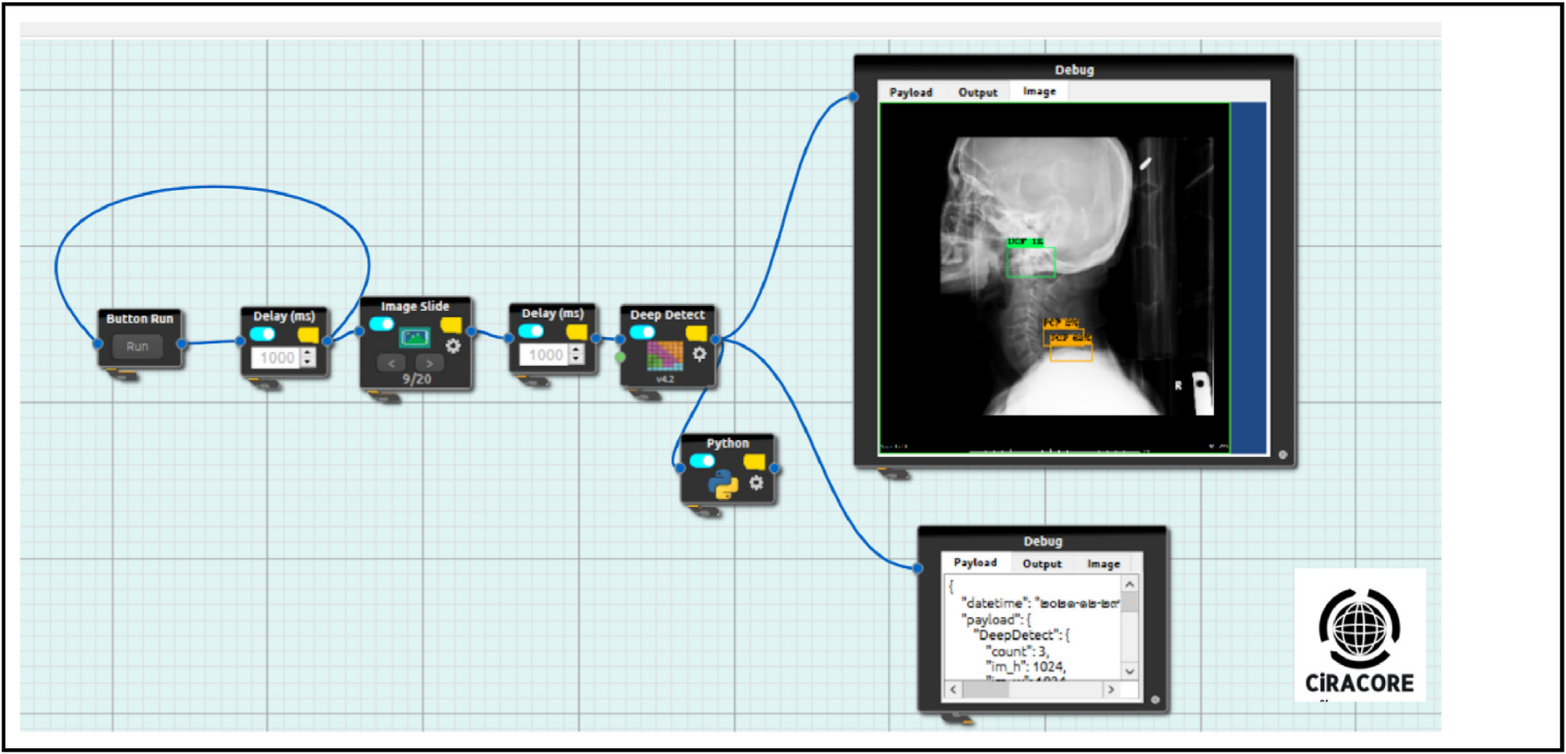
The figure shows drag and drop boxes with node-flow programming style provided by CiRA CORE Deep learning platform.

The model was implemented using CiRA CORE, the codeless deep learning software developed by King Mongkut's Institute of Technology Ladkrabang (KMILT; Figure 3). A trained model with a mini-batch size of 64 (min/max) and involving 8 subdivisions was used, with momentum and decay of 0.9 and 0.0005, respectively. The learning rate was 0.002.

The performance of the deep learning network models was assessed using an operating characteristic curve (ROC). The ROC analysis was performed using the SPSS software.

### 2.4 Statistical analysis

Continuous data were analyzed using Pearson's Chi-square test or Fisher's exact test, as well as the Mann-Whitney U test.

Simple logistic regression was employed in univariate analysis and multiple logistic regression was utilized with backward stepwise selection of variables in multivariate analysis.

### 2.5 Testing the model

The model with the best performance was determined using 40 unseen radiographs (20 negative and 20 positive). The results were evaluated for confusion matrix, sensitivity, specificity, and accuracy. For cases with positive prediction, the intersection over union scores (IoU) were calculated.

The test cases were also evaluated by a radiologist with nine years' experience, an orthopedics with seven years’ experience and a senior radiology resident. The McNemar test was used to evaluate the noninferiority of the accuracy, sensitivity, and specificity of the model compared with the radiologist, the orthopedics, and the radiology resident.

## 3 Results

Between May 2015 and May 2020, 625 patients (447 men [71.52%] and 178 women [28.48%]) underwent CT scan of the cervical spine. The average age was 38.73 years old, with the majority under the age of 65 (86.4%). Most had abnormal brain results (68.98%; Table 1). Sex and paresthesia in the extremities were two statistically significant factors according to univariate analysis (p-values < 0.05; Table 1). However, after multiple logistic regression analysis (adjust odds ratio), sex was no longer significant (adjust odds ratio and 95% CI of 1 and 0.72 (0.44–1.15), respectively; p = 0.163). Fourteen (2.24%) of the 625 patients had no indication for imaging evaluation in the CT report according to the CCR and NEXUS criteria. Of these 14 patients, three had positive CT reports. Decreased Glasgow coma score, dangerous mechanism, and midline tenderness were the most common indications, present in 368 (58.88%), 199 (31.84%), and 73 (11.68%) patients, respectively. Paresthesia in the extremities was associated with cervical spine fractures (adjusted odds ratio: 7.02 [1.77–27.79]; p = 0.006). Presence of abnormal brain findings was not considered a factor due to the high p-value (Table 2).

**Table 1:** Differences between groups.

		Total (n = 625)	No fracture (n = 434)	Fracture (n = 191)	p-value
Sex	Male	447 (71.52)	299 (68.89)	148 (77.49)	0.028
Age	Median (IQR)	36 (21–54)	34 (21–53)	39 (21–57)	0.229
	Mean (SD)	38.73 (20.43)	38.10 (20.27)	40.16 (20.78)	
Age group	≥65	85 (13.6)	58 (13.36)	27 (14.14)	0.795
**Indication**					
Dangerous mechanism		199 (31.84)	136 (31.34)	63 (32.98)	0.684
GCS ≤13, decrease GCS		368 (58.88)	266 (61.29)	102 (53.4)	0.065
paresthesia in extremities		17 (2.72)	6 (1.38)	11 (5.76)	0.002
Not ambulatory		17 (2.72)	11 (2.53)	6 (3.14)	0.667
Acute onset of neck pain		53 (8.48)	37 (8.53)	16 (8.38)	0.951
Midline tenderness		73 (11.68)	50 (11.52)	23 (12.04)	0.852
Able to bend neck		5 (0.8)	3 (0.69)	2 (1.05)	0.644
Others		82 (13.12)	53 (12.21)	29 (15.18)	0.311
Brain findings		338 (68.98)	244 (68.93)	94 (69.12)	0.967

One hundred eighty-one patients (28.9%) had cervical spine injuries. Of those, 82 (45.3%) had upper cervical spine injury, 70 (38.7%) had a lower cervical spine injury, and 29 (16%) had both upper and lower cervical spine injury.

**Table 2:** Association between outcome and factors.

Factors	OR (95% CI)	p-value	Adjusted OR (95% CI)	p-value
**Sex**		0.029		0.163
Male	1		1	
Female	0.64 (0.43–0.96)		0.72 (0.44–1.15)	
**Paresthesia in extremities**		0.004		0.006∗
No	1		1	
Yes	4.36 (1.59–11.97)		7.02 (1.77–27.79)	

Assessing the models.

Three hundred thirty-one lateral neck or lateral cervical spine radiographs from 266 patients were obtained from a list of 625 patients who underwent CT scans after trauma. One hundred two radiographs were excluded due to inadequate field of view (68 images) and the presence of metallic instrumentation (34 images). A total of 229 radiographs (129 negative and 100 positive) were included in our study. Twenty radiographs of each class were randomly selected for inclusion in the test set.

**Figure 4:**
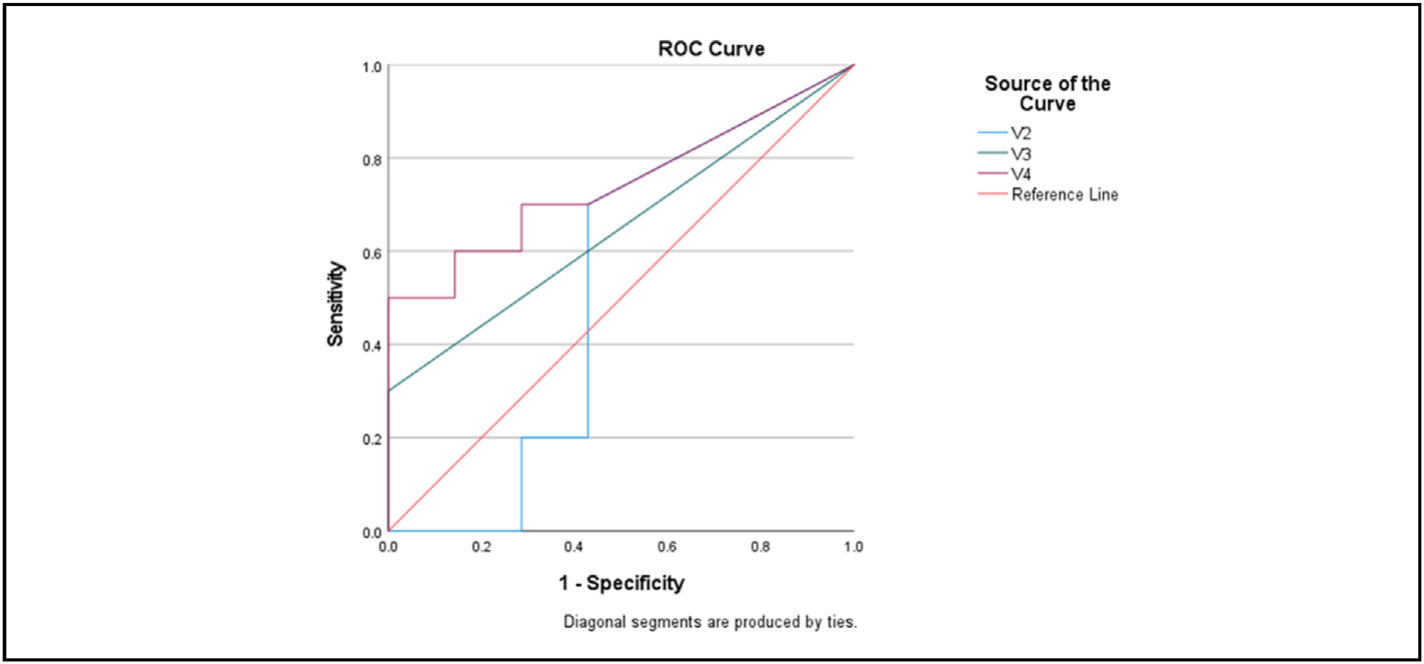
ROC curve for each model.

**Figure 5:**
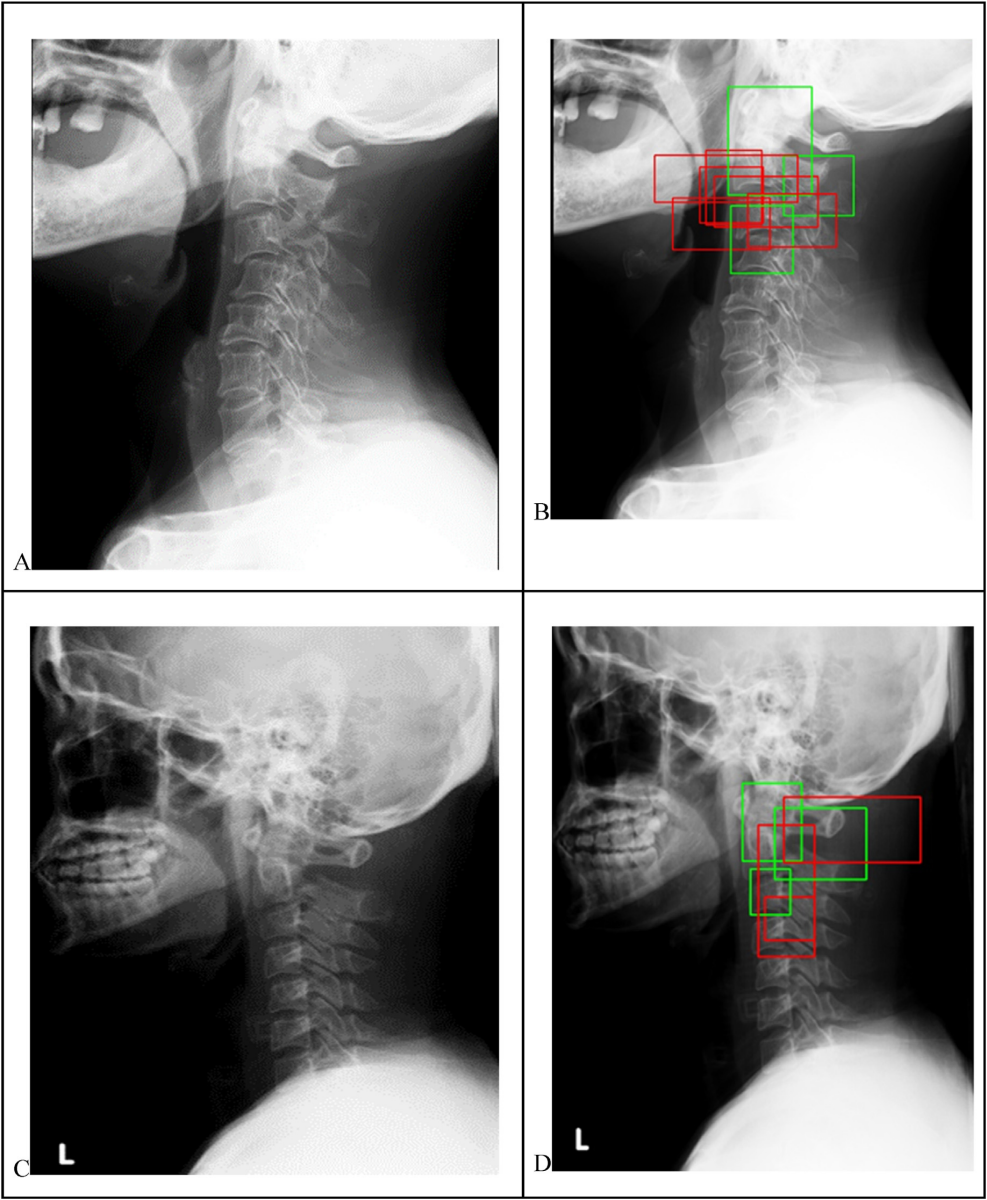
Examples of correctly labeled cases. The green bounding boxes are ground truth labels and the red boxes are the predictions. The CT reports are (A, B) C2 spinous process fracture, C3 right lamina process fracture and C3/C4 facet subluxation, (C,D) fracture at pars interarticularis of C2 vertebra and multiple linear fracture at C3 vertebral body.

The performance of the models was tested using CiRA CORE software with 40 test cases: 20 negative and 20 positive. The AUC of the ROC curve for the YOLO V4 model was higher than that of the other two (0.75 versus 0.51 and 0.65; Figure 4, Table 3). The optimum cut-off point for probability was 15%, which yielded a sensitivity, specificity, and accuracy of 80%, 72%, and 75%, respectively, using the YOLO V4 model. Mean IoU was 0.32 (0.09–0.58; Figure 5.)

**Table 3:** Area under the curve of each model.

Model	Area	Std. Error^a^	Asymptotic Sig.^b^	95% Confidence Interval	
				Lower Bound	Upper Bound
YOLO V2	.514	.164	.922	.193	.835
YOLO V3	.650	.134	.306	.387	.913
YOLO V4	.743	.121	.097	.506	.980

a Under the nonparametric assumption.

b Null hypothesis: true area = 0.5

The overall accuracy, sensitivity, and specificity of the radiologist, the orthopedics, and the radiology resident are shown in Table 4. An McNemar's test determined that the model had a higher accuracy and specificity compared with the radiology resident (p = 0.012 and 0.011, respectively). But there was not a statistically significant difference between the model and the radiologist or the orthopedics.

**Table 4:** Predictive values for the diagnosis of C-spine injury.

	Value (%)	95% CI	p-value
**Yolo V4 Model**			
Accuracy (%)	75	64.56–90.44	1
Sensitivity (%)	80	40.80–84.60	1
Specificity (%)	72	68.30–98.8	1
**Resident**			
Accuracy (%)	50	34.50–65.50	0.012∗
Sensitivity (%)	50	27.20–72.80	0.317
Specificity (%)	50	27.20–72.80	0.011∗
**Orthopedics**			
Accuracy (%)	72.5	58.66–86.34	0.527
Sensitivity (%)	65	40.80–84.60	>0.999
Specificity (%)	80	56.30–94.30	0.414
**Radiologist**			
Accuracy (%)	82.5	70.72–94.28	0.593
Sensitivity (%)	75	50.9–91.3	0.527
Specificity (%)	90	68.3–98.8	>0.999

## 4 Discussion

Lateral neck radiographs have been reported as having a high false-negative rate and limited use in screening protocols [12, 17, 18]. However, other reports have found high negative rates of cervical CT scans in trauma settings, indicating the possibility that such scans were excessive, resulting higher-than-necessary costs [10, 19, 20, 21].

In our study, 71% of CT scans of the cervical spine were negative, which amounts to approximately 89 excessive scans per year. Plain radiographs require highly experienced eyes to make an accurate diagnosis and immediate expert opinion is not always available. Our results show that deep learning can enhance the use of lateral c-spine or neck radiography with an accuracy of 75% which may be as high as the detection accuracy of expert's diagnosis. We believe that this can help physicians in terms of promptly triaging and reducing excessive CT scans.

Based on the CT reports, we found that 14 patients (2.2%) had no indication for imaging evaluation according to the CCR and NEXUS criteria [14, 22] for imaging evaluation. Of these, three had positive CT reports. About 98% of the patients had at least one indication, of whom only 29.3% (178 of 611) had cervical spine injury (either significant or non-significant). Although some information may have been missing, as our data mining did not emphasize clinical information and was based purely on CT reports, this raises concerns about the effectiveness of the CCR and NEXUS criteria and suggests a possible need for revision. For example, giving each data point a different weight as in TI-RADS might add more value to the criteria [23].

One strength of our study is that it is one of the first to use deep learning in cervical spine injury to assess plain radiographs. The results are promising in that they indicate a possible 85% reduction in excessive CT scanning (17 of 20 cases).

However, this study also had several limitations. Due to the high percentage of inadequate radiographs according to standard C1-T1 coverage, we were forced to adjust our exclusion criteria in order to have sufficient data. However, this data—although imperfect—might be a good representation of real-world data. Second, although the results are promising, we believe that better results can be obtained with more data for training. Finally, we did not classify injuries according to standard classification which would be helpful for the decision-making process for proper management [24]. The model was only trained on the detection of the injury, but different kinds of injuries have different protocols. Therefore, injuries detected by the model should be re-evaluated by a clinician or a radiologist to confirm the diagnosis.

Deep learning can improve the accuracy of lateral C-spine or neck radiographs. We believe that our results will be useful for rapid screening, especially in cases where specialists are unavailable to provide an immediate opinion. We anticipate that this will assist clinicians in quickly triaging patients and minimize the number of unnecessary CT scans.

**What is already known on this topic**

- Plain radiographs are readily available, but their interpretation requires a highly experienced clinician. Cervical spine CT has higher value but equivocal cost-effectiveness.
- Deep learning can increase the diagnostic accuracy of many imaging modalities.

**What this study adds**

- Deep learning can enhance the usefulness of plain radiographs and potentially reduce unnecessary CT scans. Nowadays, there are various deep learning platforms that require minimal coding skill, making them powerful tools for researchers.

**How this study might affect research, practice, or policy**

- The model provides a rapid interpretation of plain radiograph results, which can help clinicians evaluate patients with suspected cervical spine injury and potentially reduce unnecessary CT scans.

## Declarations

### Author contribution statement

Arunnit Boonrod: Conceived and designed the experiments; Performed the experiments; Analyzed and interpreted the data; Wrote the paper. Artit Boonrod: Conceived and designed the experiments; Analyzed and interpreted the data; Wrote the paper. Atthaphon Meethawolgul: Contributed reagents, materials, analysis tools or data; Wrote the paper. Prin Twinprai: Analyzed and interpreted the data; Wrote the paper.

### Funding statement

This work was supported by Khon Kaen University’s Research and Graduate Studies (RP 291/2564).

### Data availability statement

Data will be made available on request.

### Declaration of interests statement

The authors declare no conflict of interest.

### Additional information

No additional information.
